# Interactions between metabolic, reward and cognitive processes in appetite control: Implications for novel weight management therapies

**DOI:** 10.1177/0269881117736917

**Published:** 2017-10-26

**Authors:** Suzanne Higgs, Maartje S Spetter, Jason M Thomas, Pia Rotshtein, Michelle Lee, Manfred Hallschmid, Colin T Dourish

**Affiliations:** 1 School of Psychology, University of Birmingham, Birmingham, UK; 2 Department of Psychology, Aston University, Birmingham, UK; 3 Department of Psychology, Swansea University, Swansea, UK; 4 Institute for Medical Psychology and Behavioural Neurobiology, University Tübingen, Tübingen, Germany; 5 German Center for Diabetes Research (DZD), Tübingen, Germany; 6 Institute for Diabetes Research and Metabolic Diseases, University of Tübingen, Tübingen, Germany; 7 P1vital, Wallingford, UK

**Keywords:** Food reward, cognition, metabolic signals, appetite control

## Abstract

Traditional models of appetite control have emphasised the role of parallel homeostatic and hedonic systems, but more recently the distinction between independent homeostatic and hedonic systems has been abandoned in favour of a framework that emphasises the cross talk between the neurochemical substrates of the two systems. In addition, evidence has emerged more recently, that higher level cognitive functions such as learning, memory and attention play an important role in everyday appetite control and that homeostatic signals also play a role in cognition. Here, we review this evidence and present a comprehensive model of the control of appetite that integrates cognitive, homeostatic and reward mechanisms. We discuss the implications of this model for understanding the factors that may contribute to disordered patterns of eating and suggest opportunities for developing more effective treatment approaches for eating disorders and weight management.

## Introduction

Greater understanding of the mechanisms underlying appetite control is crucial to address the health problems that are associated with poor dietary choices and overconsumption of food (Wang et al., 2011). Moreover, given the health costs associated with unhealthy eating patterns (Scarborough et al., 2011), it is important to explore new avenues for improving eating behaviour through the development of comprehensive models of appetite control that open the way for thinking about novel interventions and advice on nutrition.

The neural control of eating involves activity in brain circuits that process signals of nutritional state and food reward value. The ingestion of food reduces the incentive value of food, which is reflected in decreased activity in reward-related brain areas (Spetter et al., 2012; Thomas et al., 2015). However, eating is also influenced by higher cognitive processes such as attention and memory (Higgs, 2016) and it has recently been suggested that metabolic signals may have indirect effects on food reward processing via alterations in higher cognitive function (Thomas et al., 2014). This review will highlight new evidence that the control of eating involves interactions between cognitive, metabolic and reward mechanisms. We will consider how this new framework can inform our understanding of the causes of overeating and comorbidities between cognitive dysfunction and disordered eating. Finally, we will assess the implications for the development of new approaches to healthy eating and weight management.

## Concepts in appetite control

Traditionally, appetite has been investigated by two parallel lines of research focusing on the homeostatic and hedonic systems. Research on the neurobehavioural control of appetite, by homeostatic mechanisms, has focused for many years on the role of nutrient sensing processes coordinated in the brain by the hypothalamus (Waterson and Horvath, 2015). This research has been important in identifying how information about metabolic state (for example, information about whether we are fed or fasted) reaches the brain from the periphery and then undergoes further processing so that eventually motor outputs (eating behaviours) are generated. It is well known that the metabolic signals generated by the gastrointestinal (GI) tract when food is ingested are associated with changes in eating behaviour. Specifically, hormones including cholecystokinin (CCK) and glucagon-like peptide 1 (GLP-1) are released when food is eaten and this is associated with reductions in intake (Antin et al., 1975; Turton et al., 1996). Conversely, ghrelin, a hormone released from the stomach during fasting, is associated with increased food intake (Nakazato et al., 2001).

Appetite is also known to be responsive to hormones such as the pancreatic hormone insulin and the adipokine leptin that are secreted in proportion to the amount of fat stored in adipocytes (Halaas et al., 1995; Woods et al., 1979). The arcuate nucleus (ARC) of the hypothalamus acts as an integrator of such signals from the periphery. Pro-opiomelanocortin (POMC)/cocaine, amphetamine-regulated transcript (CART) and agouti-related protein (AgRP)/neuropeptide Y (NPY) neurones in the ARC have been strongly implicated in the control of food intake (for reviews see Clemmensen et al., 2017; Waterson and Horvath, 2015; Yeo and Heisler, 2012). These hypothalamic neurones express receptors for leptin and ghrelin and are modulated by the neurotransmitter serotonin (5-HT; for review see Garfield and Heisler, 2009). The caudal brainstem is another major integrator of information on nutrient ingestion relayed from the gut (for review see Grill and Hayes, 2012). Neurones in the nucleus tractus solitarius (NTS) are responsible for processing multiple nutrient status signals from the periphery and relay output to other regions involved in the control of intake including the hypothalamus (Grill and Hayes, 2012).

From a hedonic system perspective, research has focused on the importance of reward processes in motivated behaviours, including eating. This research has elucidated how cues associated with the consumption of tasty foods can promote food seeking and intake (Berridge, 1996). When we eat a food that evokes a pleasurable hedonic response, we will come to associate the characteristics of that food (e.g. the sight and the smell of the food) with the positive consequence (‘liking’ response). As a result of this learning, the food-associated visual and olfactory cues acquire the ability to become sought after (they become ‘wanted’) (Berridge, 1996). For example, we might have a strong desire to consume pizza if we see a shop advertising pizza from which a strong smell of pizza is emanating.

The neurobiology of food reward circuitry has been well studied: coordinated activity in a network of opioidergic and cannabinoidergic hedonic hotspots in the nucleus accumbens (NAcc), ventral pallidum and brainstem is thought to mediate ‘liking’ responses (e.g. Higgs et al., 2003; Higgs and Cooper, 1996; Mahler et al., 2007; Pecina and Berridge, 2005; for a review see Castro and Berridge, 2014). On the other hand, evidence suggests that the mesolimbic dopamine neurotransmitter system is crucial for food ‘wanting’ (e.g. Pecina et al., 2003; Tindell et al., 2005; Wyvell and Berridge, 2000; for a review see Castro and Berridge, 2014).

More recently, the idea that there are independent homeostatic and hedonic systems has been abandoned in favour of a framework that emphasises the crosstalk between the neurochemical substrates of the two systems (Berthoud et al., 2017). This approach is consistent with incentive motivation theories of behaviour, which argue that metabolic state influences eating behaviour by modulating the hedonic value of food and food-associated cues (Toates, 1986). It is also consistent with evidence that pleasurable sensations are affected by metabolic state, a process known as alliesthesia (Cabanac, 1971, 1979). Food is more highly liked and desired when hungry and less liked when satiated: the smell and taste of a pizza is usually less alluring when we have just eaten (Berridge et al., 2010).

## Metabolic signals modulate food reward circuitry

Extensive evidence has now accumulated that neural systems of food reward interact with homeostatic networks, thus providing a mechanism by which food deprivation or satiation affects food attractiveness. For example, food deprivation increases the incentive value of food, which is reflected in enhanced responses to appetitive stimuli in reward-related brain areas in humans (Cornier et al., 2009; DelParigi et al., 2005; Führer et al., 2008; Gautier et al., 2000; Goldstone et al., 2009; Haase et al., 2009; Killgore et al., 2003; Kringelbach et al., 2003; LaBar et al., 2001; Porubska et al., 2006; Simmons et al., 2005) whereas satiation decreases responses in reward-related circuitry (Fletcher et al., 2010; Thomas et al., 2015). These effects are likely to be mediated by a direct action of metabolic signals, such as leptin, insulin, GLP-1 and ghrelin, on the mesocorticolimbic dopamine system (Batterham et al., 2007; Farooqi et al., 2007; Figlewicz et al., 2006; Fulton et al., 2006; Guthoff et al., 2010; Hallschmid et al., 2012; Jerlhag et al., 2012; Malik et al., 2008). It is well known that insulin acting at peripheral sites promotes bodyweight gain by stimulating energy storage. However, specific stimulation of brain insulin receptors decreases activity in mesolimbic dopamine circuits and reduces food reward (Figlewicz, 2003; Mebel et al., 2012), probably because insulin also functions as a negative feedback signal to the brain about levels of body fat (Woods et al., 1979). Hence, insulin may mediate reduced reward after consumption of high-energy meals (Davis et al., 2010). In line with this suggestion, we found that intranasal administration of insulin to healthy humans reduces the intake of palatable food in the post-prandial state (Hallschmid et al., 2012). Leptin administration also decreases activity in the mesolimbic dopamine system of rats (Fulton et al., 2006) and leptin replacement in humans with a congenital absence of leptin reduces heightened reward responses to food pictures when satiated (Farooqi et al., 2007). There have also been recent advances in our understanding of the role of reward-related mechanisms in the effects of GLP-1 signalling on eating behaviours from rodent studies (Alhadeff et al., 2012; Dickson et al., 2012; Dossat et al., 2011). GLP-1 receptor activation in the ventral tegmental area (VTA) and NAcc core reduces intake of highly palatable, energy dense food without affecting intake of a standard diet (Alhadeff et al., 2012). These data suggest that GLP-1 signalling in the mesolimbic system may have a selective effect to reduce the rewarding value of palatable food. In support of this suggestion, it has been reported that the GLP-1 analogue liraglutide reduces activity in brain reward circuitry in participants with type 2 diabetes (Farr et al., 2016). Conversely, the orexigenic hormone ghrelin stimulates dopaminergic (DA) activity (Jerlhag et al., 2012) and increases responding for sucrose in rats when injected peripherally and directly into the VTA (Skibicka et al., 2012a,b). Ghrelin has also been found to increase the neural response to food pictures in reward-related circuitry (orbitofrontal cortex (OFC) and striatum) in humans (Malik et al., 2008).

Investigations of the role of 5-HT in the control of appetite have focused on both homeostatic and hedonic mechanisms (Blundell, 1984; Dourish, 1995). Neurobiological studies have demonstrated the role of hypothalamic mechanisms in the effects of serotonergic drugs on food intake. The melanocortin system of the ARC has been identified as a key network in the anorectic effects of 5-HT agonists, including the 5-HT_2C_ receptor agonist lorcaserin (Heisler et al., 2002, 2006; Sohn et al., 2011), which has recently been approved by the US Food and Drug Administration (FDA) for weight management. Alterations in 5-HT transmission also affect reward-related circuits in the brain to influence food intake. Thus, 5-HT_2C_ receptors expressed in the VTA (Bubar and Cunningham, 2007) modulate activity of DA projections to the NAcc to alter motivation for food and drug reinforcers in rats (Fletcher et al., 2004; Higgins et al., 2013). These preclinical data suggest a specific role for 5-HT_2C_ receptor activation in linking hypothalamic energy-sensing mechanisms to motivational aspects of eating behaviour. Recently we reported that the 5-HT_2C_ receptor agonist meta-chlorophenylpiperazine (mCPP) reduced consumption of a palatable energy dense snack eaten after a satiating meal in healthy volunteers. Using functional magnetic resonance imaging (fMRI) we further observed that mCPP caused a marked reduction in activity across reward-related brain regions in response to the sight of food pictures. These data suggest a role for 5-HT_2C_ receptor mechanisms in inhibiting food-reward, especially after eating.

In addition to direct links between metabolic signalling and the mesolimbic dopamine system, there are indirect links via the lateral hypothalamus (LH) (Leinninger et al., 2009). It is well established that electrical stimulation of the LH elicits feeding in rats (Hoebel and Teittelbaum, 1962; Valenstein et al., 1968) and that this effect is modulated by metabolic state (Sheng et al., 2014). There is now evidence that these effects are mediated by heterogeneous projections from the LH to the VTA, including neurones expressing orexin (Harris et al., 2005), neurotensin (Leinninger et al., 2009), and gamma-aminobutyric acid (GABA) or glutamate (Nieh et al., 2015, 2016). In addition, recent evidence suggests a role for agouti-related peptide (AGRP)/neuropeptide Y (NPY) neurones in the ARC in integrating internal metabolic signals with external signals on food availability to provide an output that drives downstream reward circuitry and promotes eating in mice (Chen et al., 2015).

The effects of metabolic signals on food reward go some way to explaining why food is usually more attractive when we are hungry and less attractive when we are full. But individuals do not always respond to the presence of food cues by initiating eating, and eating may continue even when someone has already consumed a large amount of food. Eating is a complex behaviour that can be initiated or brought to a close depending on a multitude of influences that include taste and smell as well as contextual factors and prior experiences (Higgs, 2005). Individual differences in the initial response to a food cue, sensitivity to metabolic signals, and cognitions will affect the outcome. As such, eating may be inhibited even in the presence of highly palatable food-relevant stimuli or a depleted metabolic state. To provide a simplified example we can think about a situation in which we are confronted with a food cue, such as the sight, smell or taste of food or a food advert. An appetitive response to this stimulus may be inhibited if someone has a desire to avoid consumption of certain foods bearing in mind long-term consequences for health. This kind of thinking has led to an expanded view of the neural control of appetite that includes brain regions important for learning, memory and attention including the hippocampus, amygdala and pre-frontal cortex (for reviews see Coppin, 2016; Hargrave et al., 2016; Kanoski et al., 2017; Parent, 2016).

## Cognitive modulation of food reward

**Figure 1.**
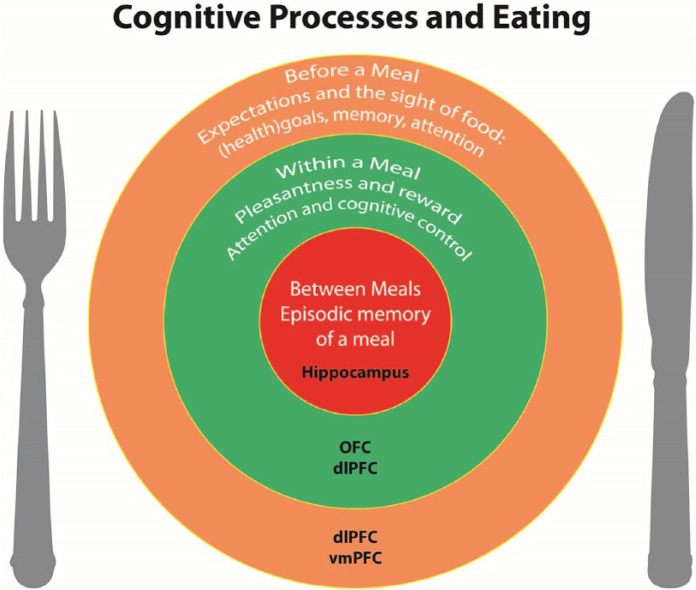
Cognitive processes throughout the day that influence eating behaviour. The outer circle provides an overview of the processes that operate before a meal: the expectation and sight of the food to be consumed, and the interplay between any (health) goals, memory of the taste and pleasure of the food and attention to food cues will determine if individuals will start eating and what kind of food they will choose. The middle circle represents within-meal processes that influence the amount consumed: pleasantness and reward values will decrease while eating and ultimately lead to meal termination. Additionally, attention to the process of eating and cognitive control also will influence the termination of the meal. The inner circle represents the processes operating between meals, for example episodic memory of a meal will influence decisions about when to eat a next meal. dlPFC: dorsolateral prefrontal cortex; OFC: orbitofrontal cortex; vmPFC: ventromedial-prefrontal cortex.

The view that motivation to eat depends on cognitive modulation of reward processes is gaining traction and it has been argued that everyday control of appetite involves cognitive processes such as learning, attention and memory (Higgs, 2016). For example, it has been demonstrated that a focus on the longer-term health outcomes of eating unhealthy foods is associated with inhibition of reward-related brain activity (Hare et al., 2009, 2011a). These cognitive processes are likely to operate across all aspects of appetite control including before a meal begins, during a meal and in the intervals between meals (see Figure 1) and will be reviewed briefly here. One point of note is that suggesting eating involves cognition does not imply that we consciously consider food decisions all the time. Much of the time, eating seems to engage no mental effort but this does not infer that eating is ‘mindless’ (Herman and Polivy, 2014). There are other complex behaviours, such as driving a car, that we would readily accept involve the coordination of complex cognitive processes including attention, learning and memory but also appear routine and/or effortless. Thus we can think of eating in the same way: we may be made aware of the underpinning mental processes but they are not constantly in awareness.

## Cognitive processes involved in responses to food cues before eating begins

The sight of a tasty food can elicit appetitive behaviours, such as food seeking and a desire to eat, and it will also evoke cognitive expectations about how the food will taste, how satiating it is and whether eating it will be consistent with our longer-term health goals (Brunstrom, 2011; Rangel, 2013). These expectations are factored into choices about whether to eat and/or how much to eat (Rangel and Hare, 2010). They are based on conditioned responses that arise from learned associations about the consequences of eating (Dickinson, 2012) as well as mental simulations of the outcomes of specific choices based on episodic memories (Daw and Shohamy, 2008; Lengyel and Dayan, 2008). The value of individual predicted outcomes is computed in the ventromedial-prefrontal cortex (vmPFC) and a network that includes dorsolateral prefrontal cortex (dlPFC) uses the value input from the vmPFC to select an action (Hare et al., 2009, 2011b). This system enables eating behaviour that is goal directed, and adaptable to circumstance, rather than simply food cue driven. Thus, if we have a long-term goal of healthy eating, then an urge to consume a tempting food that is energy dense, but nutritionally deplete, may be resisted. Alternatively, if we have a positive memory of eating a food in a specific restaurant, then this might bias our decision towards choosing that food (Robinson et al., 2012).

However, one’s ability to maintain goal-directed behaviour will be affected by several factors including: whether the longer-term consequences of behaviour are retrieved from memory and are then a focus of attention (Hare et al., 2010; Hofmann et al., 2012; Whitelock et al., 2017); the extent to which we are exposed to stimuli that trigger competing cue-driven urges in our food environment; and whether other competing cognitive demands like watching television interfere with the ability to inhibit these competing responses (Braude and Stevenson, 2014; Ward and Mann, 2000), which may explain why dieting often fails (Herman and Mack, 1975; Herman and Polivy, 2004).

There are also individual differences in the ability to adhere to longer-term goals in the face of immediate rewards. The conflict between the delayed rewards of good health versus the immediate reward of a tasty food is a dilemma modelled in the delay discounting task (McHugh and Wood, 2008). In this task, participants are presented with a choice between a small reward available immediately, or a larger reward available after a delay. The indifference point (IP) is the value at which the participant is indifferent to the reward being received now or after a delay. A low IP value indicates that the participant is not very willing to wait for the reward: in other words they discount the future reward value. Discounting of the future on both money and food-based tasks has been related to over eating and obesity (Bickel et al., 2014; Jarmolowicz et al., 2014; Price et al., 2016; Weller et al., 2008). A key factor in delay discounting is likely to be the ability or lack of ability to inhibit pre-potent responses, which has also been linked to obesity and overconsumption of palatable foods (Hall, 2012; Hofmann et al., 2009; Nederkoorn et al., 2006). Hence, cognitive processes of inhibitory control, most likely underpinned by activity in the dLPFC (e.g. Ballard and Knutson, 2009), are also involved in the response to food cues (Higgs, 2016).

The desire to eat may be triggered by the sight of food, but also by thoughts of food that come spontaneously to mind, especially if one is hungry (Berry et al., 2007). Whether or not we notice food around us, or bring food easily to mind, is influenced by higher-level cognitive processes, in particular, working memory. If we are thinking about food (holding food information in working memory), this guides our attention towards food-related stimuli in the environment (Higgs et al., 2012; Rutters et al., 2015), ensuring that food cues are likely to have a greater influence on individuals who are thinking about food; for example, individuals who are hungry (Mogg et al., 1998). Attentional bias to food cues has also been linked to increased food intake and hunger (Field et al., 2016). The underlying mechanisms are unclear but one possibility is that paying attention to a stimulus increases the readiness to execute actions associated with that stimulus e.g. reaching for a tempting food (Anderson, 2017; Krebs et al., 2010). Another possibility is that selective attention to sensory/hedonic attributes of food biases choice towards food consumption because these attributes of food are weighed more strongly than longer-term goals in reward valuation processes (Werthmann et al., 2016).

Thoughts of food may guide attention to food and stimulate appetitive behaviour but we may also experience food cravings and emotional responses if an initial thought is embellished in memory (Kavanagh et al., 2005). For example, the sight of a cookie might elicit specific memories of past eating, as discussed previously, but also recall the smell and taste of cookies and how it would feel if one ate a cookie (Papies, 2013). There is some evidence to suggest that brain areas associated with food taste are activated in response to the viewing of food pictures, suggesting that processing of food cues is grounded in the same brain areas that underpin sensory responses to food itself (Chen et al., 2016). Maintenance of this kind of elaborated food imagery in working memory most likely serves a function to facilitate food seeking in the absence of direct contact with specific cues (Kavanagh et al., 2005). However, a conscious preoccupation with food or vivid, intrusive thoughts about food may serve to bias attention towards food cues even when they have been devalued, for example in a state of satiety. Thus, a failure to inhibit intrusive thoughts about food could result in a reduced ability to dampen responses to food cues when satiated, which may cause overeating in the absence of hunger and contribute to disordered eating patterns (Higgs, 2016; Martin and Davidson, 2014).

## Cognitive processes involved in responses to food cues during eating and satiation

In the later phases of a meal, there is a decline in the perceived pleasantness of food that contributes to the cessation of eating (Hetherington, 1996). The reduction in the rewarding properties of food as it is eaten may be specific for that food, as in sensory specific satiety (Rolls et al., 1981), but there is also a general decline in the attractiveness of all foods as mentioned previously, which is known as alliesthesia (Cabanac, 1971, 1979). Habituation of neural responses in the OFC, which codes for a representation of the reward value of the taste of food, is one mechanism that is likely to underlie within-meal reductions in food pleasantness (Critchley and Rolls, 1996; O’Doherty et al., 2000), alongside reduced signalling in the mesolimbic dopamine system. We investigated the neural underpinning of natural satiation in humans using fMRI (Thomas et al., 2015). In line with previous data on sensory specific satiety and alliesthesia, we found that eating to fullness after a natural inter-meal interval was accompanied by decreases in reward-related brain activations in the OFC and the mesocorticolimbic dopamine system. A novel finding was that natural satiation increased activity in the dorsolateral prefrontal cortex (dlPFC) (Thomas et al., 2015), an area that is associated with attention, memory and cognitive control (Duncan, 2013). Moreover, activity in the vmPFC was negatively correlated with activity in the dlPFC and connectivity between these areas was increased in the satiated state. These data suggest that natural satiation is associated with a distributed pattern of changes in neural activity suggestive of metabolic influences on both reward-related circuitry and areas involved in higher cognitive functions and decision making. An implication of this finding is that if either habituation or reward valuation processes are disrupted, then satiation will be impaired, as has been observed for eating while distracted (Braude and Stevenson, 2014). The specific cognitive processes involved have yet to be elucidated but may relate to the role the dlPFC plays in modulating food value in response to changes in context (in this case metabolic state) (Rudorf and Hare, 2014). Alternatively, given the importance of the dlPFC for working memory, there may be an important role for working memory modulation of attention to food cues (Curtis and D’Esposito, 2003).

## Cognitive processes involved in responses to food cues in intervals between eating episodes

Cognitive processes are also important for the inhibition of food intake that occurs after an eating episode (satiety). There is considerable experimental evidence that memory of a recent eating episode inhibits eating (Higgs, 2016). A striking example of the importance of memory for recent eating in satiety is that amnesic patients who are unable to recall recent eating will eat multiple meals in quick succession (Hebben et al., 1985; Higgs et al., 2008b; Rozin et al., 1998). Manipulation of the memory of a meal in healthy volunteers is also sufficient to affect snacking after that meal. Enhancing memory of recent eating by facilitating recall or augmenting encoding of food memories decreases subsequent food intake (Higgs, 2002; Higgs et al., 2008a; Higgs and Donohoe, 2011; Robinson et al., 2014). On the other hand, if encoding of episodic food memories is disrupted by engagement in a secondary activity, such as watching television or playing a computer game while eating, subsequent snack intake is increased (Higgs, 2016; Higgs and Woodward, 2009; Mittal et al., 2011; Moray et al., 2007; Oldham-Cooper et al., 2011). Moreover, remembered food intake is a better predictor of later hunger than the amount eaten (Brunstrom et al., 2012). The data from humans on the importance of meal memories in satiety is supported by evidence that hippocampal-dependent episodic memory of a recently eaten meal influences the timing of the next meal and the amount consumed at that next meal in rats (Parent, 2016). Rats with selective lesions to the hippocampus have disturbed meal patterns and overeat (Clifton et al., 1998; Davidson et al., 2005; Davidson and Jarrard, 1993) and temporary inactivation of the hippocampus of rats accelerates the onset of the next meal (Henderson et al., 2013). Taken together, these data suggest that satiety is in part cognitively constructed and dependent upon episodic memory (Higgs, 2008; Redden, 2014).

## Linking cognitive processes of appetite control with metabolic signalling: the role of hormonal and neurotransmitter mechanisms

Until recently, research on the cognitive control of eating had not been well integrated with research on metabolic control. An emerging literature is documenting the broader effects of metabolic signals on higher-level cognitive processes such as attention and learning and memory. This literature suggests that some effects of metabolic signals on eating may be mediated by their effects on cognition, although research specifically linking cognitive effects of metabolic signals with appetite is in its infancy.

## Insulin and cognition

A high density of insulin receptors is expressed in the cerebral cortex, olfactory bulb, hippocampus, cerebellum and hypothalamus (Unger et al., 1991). Intracerebroventricular administration of insulin to rodents and intranasal insulin administration to humans (at doses up to 80 IU) raises brain insulin levels without inducing concomitant changes in blood insulin or glucose levels and improves memory (e.g. Benedict et al., 2004; Park et al., 2000). There is substantial evidence that centrally acting insulin enhances cognitive function (Shemesh et al., 2012). For example, intranasal insulin improves declarative memory and working memory in humans (Benedict et al., 2004, 2008; Hallschmid et al., 2008, 2012; Stockhorst et al., 2004). Neural responses to intranasal insulin and resting state function have been examined using fMRI and the results are consistent with the idea that insulin acts to alter neural activity in brain circuits that are important for higher cognitive function, including the pre-frontal cortex (e.g. Kullmann et al., 2013, 2015). In addition, the results of clinical trials of the effects of intranasal insulin in patients with either mild cognitive impairment (MCI) or Alzheimer’s disease suggest improvements in verbal and visuo-spatial working memory in these patients (Claxton et al., 2015; Reger et al., 2008a,b). Furthermore, central nervous system (CNS) insulin resistance has been linked to cognitive impairment (Craft et al., 2013; De Felice, 2013), including reduced performance on tests of episodic and working memory (Talbot et al., 2012) and impaired performance on an episodic memory task that is linked to reduced activity in the core neural network associated with memory recall (Cheke et al., 2017). Insulin resistance is also a risk factor for the development of dementia (Chatterjee et al., 2015). The specific mechanisms underlying the effects of CNS insulin administration and insulin resistance on memory have yet to be fully elucidated, but it is likely that regulation of synaptic plasticity in the hippocampus is involved (Fadel and Reagan, 2016).

In relation to the effects of enhanced brain-insulin signalling on food intake (Guthoff et al., 2010; Hallschmid et al., 2010), it is unclear to what extent its pro-cognitive effects play a role. Data from a study by Hallschmid and colleagues (2012) suggest that insulin enhancement of consolidation of a recent meal memory is not a likely mechanism, but the possibility remains that the effects of insulin in the hippocampus may mediate encoding of meal memories. Whether effects of meal-related insulin secretion on working memory are involved in active inhibitory processes of context dependent reward valuation that may occur towards the end of a meal is currently being explored in our laboratory.

## Leptin and cognition

Leptin receptors are located in the cerebral cortex, hippocampus, basal ganglia, hypothalamus, brainstem and cerebellum (Elmquist et al., 1998; Hâkansson et al., 1998; Savioz et al., 1997; Shanley et al., 2002) and there is evidence that leptin has effects on cognition (Farr et al., 2015; Morrison, 2009). At the cellular level, leptin plays a role in the synaptic plasticity of hippocampal neurones as well as long-term potentiation (LTP) (Harvey et al., 2005; Irving and Harvey, 2014). Leptin administration has been reported to improve memory function in rodents (Farr et al., 2006; Oomura et al., 2006), whereas cognitive performance is impaired in genetic models of leptin deficiency (Li et al., 2002; Paz-Filho et al., 2008). As with insulin, leptin resistance is also associated with impaired cognition, especially during aging, and impaired leptin function may contribute to cognitive impairment in MCI in humans (Holden et al., 2009; Witte et al., 2016).

Interestingly, while leptin’s effects on cognition have not been directly linked to food intake in humans, leptin replacement has been reported to reduce neuronal activity to food images in the insular, parietal and temporal cortex but increase activation in the prefrontal cortex (Baicy et al., 2007), suggesting a potential role for leptin in inhibitory cognitive processes related to satiation (Thomas et al., 2015). In addition, administration of leptin to the ventral hippocampus of rats suppressed both food intake and memory consolidation for the spatial location of a food reward (Kanoski et al., 2011). These data suggest that the effects of leptin on eating may be mediated in part by its effects on the retrieval of food memories. Further investigation of the relationship of the pro-cognitive effects of leptin to appetite control are warranted.

## GLP-1 receptors and cognition

Activation of either peripheral or central GLP-1 receptors (GLP-1Rs) in the hypothalamus and NTS reduces food intake (Hayes et al., 2011; Holst, 2007; Schick et al., 2003). GLP-1Rs are also present in the hippocampus (Hamilton and Hölscher, 2009) and their activation improves learning and memory, including hippocampal -dependent spatial memory in the Morris water maze (During et al., 2003). Further, GLP-1R knockout mice exhibit impairment in object recognition learning (Abbas et al., 2009) and the GLP-1 agonist liraglutide enhances memory in a mouse model of Alzheimer’s disease (Hansen et al., 2015). Liraglutide is currently in clinical trials for Alzheimer’s disease (NCT01843075) but there has been little investigation of the effects of GLP-1 ligands on cognition in healthy humans.

A link between the anorectic and cognitive effects of GLP-1 receptor activation is provided by the observation that injection of the GLP-1R agonist exendin-4 into the ventral hippocampus of rats reduces meal size and lever pressing for palatable food (Hsu et al., 2015a). In contrast, GLP-1 receptor activation had no effect on the expression of a conditioned place preference (CPP) for food (Hsu et al., 2015a). A possible explanation offered by the authors is that exendin-4 may only decrease food-related responding when there is food present during the test session (in the CPP paradigm there is no food available during testing). The effects of exendin-4 in the hippocampus differ from those of leptin, since leptin reduced retrieval of food-related memories when delivered into the hippocampus. An interesting point to consider in future research will be the time course of the actions of long-term adiposity-related factors such as leptin versus short-term metabolic signals, i.e. prandial signals like GLP-1, on cognition and food intake. For example, prandial signals might be expected to have a greater influence on cognitive functions that are important for meal termination (satiation) whereas adipose factors might have a more significant role to play in cognitive mechanisms involved in meal initiation.

## 5-HT and cognition

5-HT plays in a role in modulating cognitive function, although the effects of global manipulations of 5-HT on memory and attention in healthy volunteers are generally small. Nevertheless, reducing 5-HT by acute depletion of the 5-HT precursor tryptophan produces reliable impairment of memory consolidation (Mendelsohn et al., 2009). There is also an extensive preclinical literature on the role of 5-HT receptors in cognition, in particular there has been a focus on 5-HT_1A_ receptors, 5-HT_3_ receptors and, more recently, 5-HT_6_ receptors (Glikmann-Johnston et al., 2015; Lummis, 2012; Machu, 2011; Ramírez, 2013). However, 5-HT_3_ receptor and 5-HT_6_ receptor antagonists (for example the 5-HT_6_ receptor antagonist, idalopirdine), which showed promising results in preclinical studies and early Phase 2 clinical trials for Alzheimer’s disease, subsequently failed in large Phase 3 trials (Lundbeck, 2016; Ramírez, 2013). Few studies have investigated the role of 5-HT receptors in cognition in healthy humans and to date there is no consistent evidence for involvement of specific 5-HT receptor subtypes (for review see Cowen and Sherwood, 2013). However, we have recently reported the novel finding that the 5-HT_2C_ receptor agonist mCPP enhances recall of emotional words (Thomas et al., 2014). Given that the effect of mCPP on recall was unlikely to be related to effects on anxiety, further investigation of the specific role of the 5-HT_2C_ receptor in memory function is warranted. An interesting possibility is that mCPP (and by implication the 5-HT_2C_ receptor agonist, lorcaserin, which is marketed for obesity) might act to decrease food intake via enhancement of meal memories (Thomas et al., 2014).

There is a large literature that has implicated 5-HT in behavioural inhibition (e.g. Faulkner and Deakin, 2014; Soubrié, 1985). 5-HT is thought to play a specific role in behavioural inhibition that occurs in response to predictions of aversive outcomes (Boureau and Dayan, 2011; Crockett et al., 2009, 2012; Dayan and Huys, 2009) and in the ability to wait in order to obtain future reward, a specific type of impulsive responding (Miyazaki et al., 2014). Recently, it has been proposed that these actions are captured by a framework positing that 5-HT affects cognitive processes involved in action control and value-based decision making (Cools et al., 2011; Meyniel et al., 2016). Specifically, it has been argued that 5-HT overcomes the costs of actions, such as the cost of having to wait to receive a reward, to affect action selection, perhaps by down-regulating the weight the cost has in the decision to produce an effort (Meyniel et al., 2016; Schweighofer et al., 2008). Relating this idea to food choices, 5-HT may overcome the cost associated with the delayed benefits of choosing a ‘healthy’ food, thus facilitating goal-directed food choices (Vlaev et al., 2017). In the context of food intake, 5-HT could enhance the prefrontal cortical control of food value computations that may occur during satiation (Thomas et al., 2015), although this remains to be tested.

## Ghrelin and cognition

Ghrelin is the endogenous ligand of the growth hormone secretagogue receptor (GHSR), and is highly expressed in the ARC and in the hippocampus (Bennett et al., 1997; Guan et al., 1997; Zigman et al., 2006). Ghrelin has been reported to enhance spatial learning and memory formation and promote the formation of synapses in the hippocampus in mice (Diano et al., 2006). Activation of GHSRs in the ventral hippocampus increases food intake and enhances feeding in response to external food-associated cues in rats (Kanoski et al., 2013). These data suggest a role for ghrelin signalling in the ventral hippocampus in learning about food cues to facilitate foraging behaviour (Diano et al., 2006; Hsu et al., 2015b). Consistent with this proposal, ghrelin administration to humans has been reported to enhance memory for food compared to non-food pictures in a simple recognition paradigm (Malik et al., 2008) and increase hippocampal activation while viewing food pictures (Goldstone et al., 2014). However, a recent study failed to identify an effect of ghrelin on either spatial memory encoding or consolidation (Kunath et al., 2016) and there appears to be no clear relationship between cognitive function and serum ghrelin levels (Gahete et al., 2011; Spitznagel et al., 2010; Stoyanova, 2014; Theodoropoulou et al., 2012). There is much still to learn about the potential cognitive enhancing effects of ghrelin in humans. A comparison of the effects of ghrelin, insulin and leptin and ligands for 5-HT and GLP-1 receptors in a range of behavioural and fMRI tasks might help differentiate their effects on cognition and further elucidate their role in appetite control.

In summary, activity in multiple metabolic signalling pathways is associated with alterations in cognition. While the effects of metabolic signals on cognitive performance and eating behaviour have traditionally been considered separately, it is increasingly apparent that an integrated approach may be more successful in advancing our understanding of this complex area of research (e.g. Rangel, 2013). In addition, recent results suggest that nutrition-related signals are likely to serve an important role in modulating the cognitive processes that underpin eating behaviours. However, it is important to note that much research to date has been conducted using animal models and further work is required to assess the extent to which these findings translate to humans.

## Implications for understanding and treating disordered eating

There is a growing appreciation that obesity is associated with alterations to brain structure and function that are linked with neurocognitive problems, particularly in the domains of learning and memory and decision-making (Horstmann, 2017; Prickett et al., 2015; Stoeckel et al., 2016). There is also evidence that a high-fat/high-sugar diet (the so called ‘Western diet’) can have detrimental effects on cognitive function (Hsu and Kanoski, 2014). Given what is now known about the impact of metabolic signals on cognition, it is possible that neurocognitive changes associated with obesity may result from metabolic adaptations that occur in response to obesity and the consumption of certain diets (Stoeckel et al., 2016). However, neurocognitive problems may also be a cause of obesity given data on the importance of cognitive processes for appetite control (Higgs, 2016). The vicious cycle model of obesity, metabolic disease and cognitive decline (Davidson et al., 2014) proposes that eating a high-fat/high-sugar diet may lead to changes in the brain (most likely hippocampal dysfunction) that result in greater responsiveness to food-related cues, which in turn leads to overconsumption and weight gain in a perpetuating cycle (Kanoski and Davidson, 2011). However, there is some evidence that these brain and behavioural changes may be reversible. For example, the results of a recent meta-analysis suggest that intentional weight loss is associated with improvements in cognitive function in individuals who are overweight and/or obese (Veronese et al., 2017). These data suggest that interventions targeting diet- and/or obesity-induced changes in cognition could be helpful in breaking the vicious cycle.

One approach would be to develop cognitive training programmes that strengthen the ability to inhibit responses to food. A number of such training programmes have been developed and are currently in the early stages of testing (Allom et al., 2016; Stice et al., 2016). There is some evidence that programmes aimed at altering eating behaviour by enhancing inhibitory control can decrease food intake, but the specific cognitive mechanisms underlying these effects have not yet been elucidated (Veling et al., 2017). Other potentially promising programmes have targeted working memory processes (Houben et al., 2016) or used a smartphone application (app) to target food memory recall and ‘attentive’ processes during eating (Robinson et al., 2013). An interesting approach would be to combine these cognitive interventions with dietary and surgical interventions for obesity to enhance inhibitory control of food intake. Interestingly, bariatric surgery is associated with improvements in cognitive function (Handley et al., 2016). The underlying mechanisms are not well understood but are unlikely to be explained by weight loss alone and may relate to changes in metabolic signalling soon observed soon after surgery (Handley et al., 2016). For example, increased serum leptin and ghrelin concentrations following bariatric surgery have been suggested to contribute to the observed postoperative cognitive improvements (Alosco et al., 2015). Exercise has also been linked with improvement in cognitive performance, specifically inhibitory control, which may indicate the potential for additional benefit of regular exercise on appetite control (Lowe et al., 2016).

Given that weight loss is difficult to achieve, and maintain, by changes to diet and exercise patterns alone, the use of approved pharmacotherapy, along with lifestyle changes, can be useful for chronic weight management (Bray et al., 2016). At present however, pharmacotherapy options for obesity are limited and there have been concerns over the long term efficacy and safety of drugs for weight management (Narayanaswami and Dwoskin, 2017). FDA-approved monotherapy drugs include phentermine (Adipex-P), orlistat (Xenical), lorcaserin (Belviq) and liraglutide (Saxenda). Recent developments in weight-management pharmacotherapies have focussed on drug combinations such as bupropion/naltrexone (Contrave) and phentermine/topiramate (Qsymia) that act on multiple targets within the appetite control system (Narayanaswami and Dwoskin, 2017). However, the weight loss induced by lorcaserin is relatively modest and while Qsymia is more efficacious than lorcaserin as a weight-loss agent it is associated with unpleasant side effects (Heal et al., 2012). Therefore, there is a need for improved drug therapies.

The effects of pharmacotherapies might be enhanced by cognitive interventions, especially if the mechanism of action to reduce food intake is at least in part explained by cognitive modulation, as may be the case for the GLP-1 receptor agonist liraglutide and the 5-HT_2C_ receptor agonist lorcaserin. New drugs could be developed that target the cognitive processes involved in appetite control. Interestingly, lisdexamphetamine (Vyvanse) has been marketed for a number of years for the treatment of cognitive symptoms of attention deficit hyperactivity disorder (ADHD) and has recently been approved by the FDA for treatment of binge eating disorder. It is unclear how lisdexamphetamine reduces binge eating but one potential mechanism relates to its effects on attentional processes. Future consideration in novel drug therapy for weight management could be given to combining cognitive-enhancing drugs with ligands that have complementary actions on metabolic targets.

Another approach to overcome problems with cognitive control in obesity would be to target pathologies in the brain areas that underlie those functions or target the neural mechanisms underlying cognitive control with non-invasive neuromodulation techniques. For example, transcranial direct current stimulation (tDCS) and repetitive transcranial magnetic stimulation (rTMS), or the non-invasive neurotherapeutic tool real-time fMRI (rt-fMRI) neurofeedback (for review see Bartholdy et al., 2013; Stoeckel et al., 2014; Val-Laillet et al., 2015) are being explored. The first proof-of-concept results in people who are overweight or obese on self-regulation (rt-fMRI) of the insula and amygdala (Frank et al., 2012; Ihssen et al., 2016) suggest both eating-related brain areas, and networks related to top-down control of appetite, (vmPFC-dlPFC connectivity) (Spetter et al., 2017), show promise for this approach. Similar activation patterns were observed when participants were asked to consciously regulate their desire for food by thinking about the longer-term consequences of eating (Hollmann et al., 2012; Yokum and Stice, 2013), however additional behavioural effects are still to be found. Neuromodulation of dlPFC resulted in a suppression of self-reported food craving and appetite scores (Goldman et al., 2011; Uher et al., 2005), and there is evidence that tDCS and rTMS reduce food consumption (Gluck et al., 2015; Jauch-Chara et al., 2014; Lapenta et al., 2014), while theta-burst stimulation of the area increased snack intake and craving (Lowe et al., 2014). Moreover, rTMS of the dlPFC in individuals with bulimia or anorexia reduced disease-associated symptoms such as food craving, feeling fat and feeling anxious (Van den Eynde et al., 2010, 2013). The promising results of these initial studies has generated significant interest (see for review Hall et al., 2017; Lowe et al., 2017), but the behavioural findings are not always consistent and further research is needed to more comprehensively assess the full potential of this approach (Cirillo et al., 2017) and deal with the significant challenges of translating laboratory based findings into the natural environment and the clinic.

Interestingly, there may also be a link from the gut microbiome to cognitive dysfunction (Noble et al., 2017), which suggests that interventions aimed at improving the gut microbiome could have positive effects on cognition that in turn may help to ameliorate cognitive problems associated with obesity and type 2 diabetes. A potential explanatory mechanism is that diet-induced changes in the gut microbiome in part underlie low-grade chronic inflammation associated with obesity (Bleau et al., 2015; Spyridaki et al., 2016): low-grade inflammation is known to adversely affect cognitive function (Miller and Spencer, 2014) and the hippocampus is particularly vulnerable to these effects (Hsu et al., 2015c). Diet-induced alterations in gut microbiota may also impair peripheral insulin sensitivity, which could contribute to cognitive problems (Noble et al., 2017).

Finally, there are implications for the treatment of mental illness because many psychiatric disorders including depression, anxiety, ADHD and schizophrenia are associated with disordered eating and obesity (Bulik et al., 2016; Javaras et al., 2008; Kaisari et al., 2017b; Simmons et al., 2016). Metabolic adaptations occurring as a result of weight gain are likely to exacerbate the cognitive impairments associated with psychiatric disorders such as schizophrenia (Bora et al., 2017). Hence, treatment of the metabolic disorder is likely to improve functional outcomes. In addition, further research is required to clarify the nature of the mechanisms underlying the association between psychiatric conditions and disordered eating. While there is a well-known contribution of medication to food intake patterns in psychiatric conditions (Correll et al., 2015), it is possible that core cognitive features of these conditions also contribute to disordered eating. For example, we recently identified that inattention symptoms of ADHD are associated with both binge-like eating and restrictive eating in ADHD (Kaisari et al., 2017a). It is possible that cognitive symptoms such as attentional problems and cognitive control, which cut across traditional categories of psychiatric disorder may help to explain comorbidities.

We have proposed a research framework to guide studies on disordered eating in psychiatric disorders based on the National Institute of Mental Health Research Domain Criteria Initiative (RDoC). The RDoC encourages research on dimensions of observable behaviour and neurobiology rather than a categorical, symptom-based approach to the study of mental health (Kaisari et al., 2017b). Our proposed framework comprises multi-modal, laboratory-based assessment of cognitive constructs and measures of eating behaviour in participants recruited from the community to span the range of variation in cognitive processes associated with psychiatric conditions. This dimensional approach ensures that potential confounds associated with clinical research (e.g. medication status) can be minimised. Our proposed framework enables testing for causal relationships between cognitive constructs and disordered eating because processes such as attention and cognitive control can be manipulated and effects on laboratory measures of eating assessed. A similar RDoC approach has been adopted to understand increased and decreased eating phenotypes in depression by relating symptom clusters to the neural mechanisms involved in mood-related appetite changes in the disorder (Simmons et al., 2016).

## Conclusions

**Figure 2.**
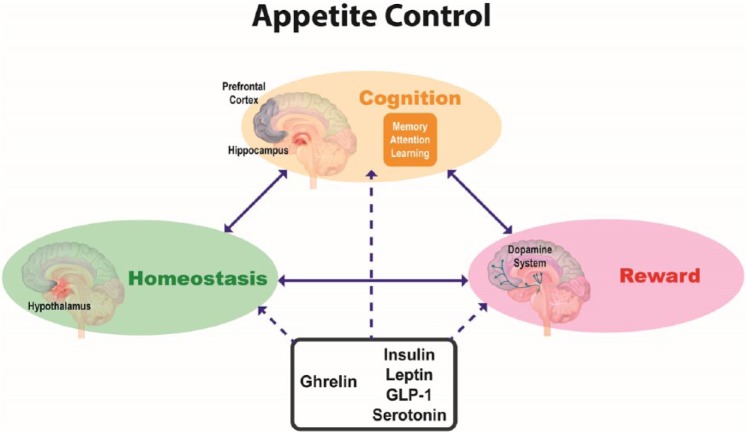
Schematic diagram outlining a model of appetite control involving interactions between homeostatic, reward and cognitive processes (indicated by solid arrows) and the modulation of these processes by metabolic signals such as insulin, leptin, glucagon-like peptide 1 (GLP-1), 5-HT and ghrelin (indicated by dashed arrows).

We have reviewed the evidence that signals relating to the ingestion of food arising from the GI tract (metabolic signals) modulate the neural homeostatic and reward processes in the brain that determine how much a food is desired. Food is less attractive when we have eaten for this reason. We have also reviewed recent evidence indicating that cognitive processes such as attention and memory underpin everyday eating behaviours. Finally, we have integrated an emerging literature on cognitive effects of metabolic signals with their effects on eating and argued that metabolic signals are likely to affect eating behaviours at least in part via modulation of higher cognitive functions. Further investigation in this area is required, in particular, to elucidate how metabolic signals influence complex food-related decision-making processes in humans. Such work will be important in fleshing out a comprehensive model of the control of appetite that integrates cognitive mechanisms with homeostatic and reward mechanisms (see Figure 2). There are important implications of this model for understanding the factors that may contribute to disordered patterns of eating. Furthermore, there are opportunities for developing more effective treatment approaches, such as combining cognitive interventions with pharmacotherapies.
